# Pore-size and polymer affect the ability of filters for washing-machines to reduce domestic emissions of fibres to sewage

**DOI:** 10.1371/journal.pone.0234248

**Published:** 2020-06-19

**Authors:** Mark Anthony Browne, Macarena Ros, Emma L. Johnston

**Affiliations:** 1 School of Biological, Earth and Environmental Sciences, University of New South Wales, Sydney, New South Wales, Australia; 2 Departamento de Biología, Facultad de Ciencias del Mar y Ambientales, Universidad de Cádiz, Puerto Real, Spain; Izmir Institute of Technology, TURKEY

## Abstract

When clothes are worn and washed, they emit fibres into the ecosystem via discharges of sewage that have been linked to the global dispersion of clothing fibres. Facilities that treat sewage divert some fibres from sewage effluent to sludge, but no current methods of filtration eliminate their environmental release. While filters for washing-machines are sold to consumers with the argument they will reduce the emissions of fibres from clothes to the environment, there is insufficient scientific peer-reviewed evidence assessing their ability to retain fibres from washed clothes and reduce environmental contamination. To improve our understanding and develop more realistic methods to assess the efficiency of filters, we washed replicate cotton and polyester garments in replicate domestic front-loaded washing-machines with and without replicate filters (micro- and milli-meter-sized pores), and then quantified the masses of the fibres retained by the filters and those released in the effluent. Here we show micrometer-sized filters significantly reduced the mass of cotton by 67% (F_2,6_ = 11.69, P<0.01) compared to effluent from appliances with no filters, whilst filters in general reduced polyester fibres in their effluent by more than 65% (micrometer-sized pores) and 74% (millimeter-sized pores) compared to effluent from appliances with no filters (F_2,12_ = 5.20, P<0.05). While filters with micrometer-sized pores caught larger masses and total proportions of fibres than filters with millimeter-sized pores, the differences were only significant for the total proportions of cotton (t = 4.799 df = 4, P<0.01). For tests with garments of either types of polymer, the filtered effluent still contained up to a third of the original masses of fibres released from the garments. Given the diversity of clothes, polymers, appliances and filters currently sold to consumers, our work shows the value of increasing the rigour (e.g. more levels of replication) when testing filters and the need for further studies that test an even greater diversity of materials and methods in order to meet the growing demand for knowledge from governments, industry and the public.

## Introduction

Globally over 50 billion apparel items per annum are reportedly made from 70 million tons of fibre [1–3]. When clothes are worn and washed, they emit fibres into the environment via discharges of sewage that have been linked to the global dispersion of clothing fibres from the Poles to the Equator and from the shallows down to oceanic depths [4–8]. Surveys suggest this contamination has increased by over 450% in 60 years [7] with more fibres found closer to large populations of humans and experiments demonstrating that fibres are continuously emitted during the washing of clothes [4,5,9]. Treatment-plants divert some fibres from sewage effluent to sludge [10] but no current filtration methods completely eliminate their release [8]. Since sewage is released to aquatic and terrestrial habitats, the contamination is widespread and is a risk to organisms far from its origin. Now clothing fibres made from plants (e.g. rayon, cotton, hemp, kenaf, flax), animals (e.g. wool, silk) and plastics (e.g. polyacrylonitrile, polyester, polyamide, polypropylene) contaminate many wild organisms [11,12].

Fibre-sized polymers are problematic because they are ingested by organisms and can accumulate in guts, [11,12] transfer to organs, tissues and cells [13,14] to induce toxicity [15,16]. Indeed, clothing chemicals and polymers can degrade the immune system of some invertebrates and kill organisms that support biodiversity through ecosystem engineering [17]. Some suggest natural fibres are less problematic [18, 19], however, some non-plastic and naturally occurring fibres can cause scar-tissue, cancer and death in humans [20, 21], while indirect toxicity of any fibre can still arise from chemicals added during manufacture, or absorbed from sewage or stormwater [17]. Despite these problems, approaches to reduce emissions of clothing fibres to the ecosystem are poorly understood, so contamination by clothing fibres is an unsolved global problem that requires robust scientific studies of how to reduce emissions [9].

Products are beginning to be sold to consumers with the argument they will reduce the emissions of fibres from clothes to the environment [22–24]. These external (e.g. Lintluv-r [25]) and internal filters (e.g. bags, balls and cylinders such as the “Guppy Friend [26]”, “Cora ball” [27], “Planet-Care” [28]) and “Filtrol [29]" are added to domestic and/or commercial washing-machines to reduce emissions of fibres to sewage. Such products are often promoted by the media [30, 31], non-government organizations [32] and companies [33, 34] as effective products for reducing emissions of fibres to environment and pollution. There is, however, insufficient scientific peer-reviewed evidence for their ability to retain fibres and reduce emissions to sewage and environmental contamination (all relevant peer-reviewed studies are summarised in Table 1) and pollution in habitats. Furthermore, a recent governmental enquiry has demanded research to reduce emissions of fibres into the environment through stewardship of products [35]. The scarcity of published evidence creates a vacuum in which misleading, confusing, or deceptive claims about the environmental benefits of products may proliferate. There are laws to help ensure consumers receive information about products that is truthful and accurate [1, 36, 37]. Developing products using scientific evidence of their efficiency would not only ensure environmental benefits but would also ensure confidence in the social license to operate is maintained between the public, government and industry. We urgently require information from robust scientific experiments to determine the capacity of technological solutions to reduce emissions of fibres to sewage that range in size from mili-, micro- and nano-meters.

**Table 1:** Summary of existing studies about emissions of fibres from clothes.

Study	Aim	Regime of wash						Textile	Metric				
		Appliance	Replicates	Detergent	Temperature (°C)	Spin (rpm)	Duration (min)	Garment	Polymer	Pore-size (μm)	Number	Mass	Length
Browne et al. [4]	Test if fibres are emitted from clothes when washed	Front	3	No	40	600	N/A	Blankets, fleeces, shirts	Polyester	1.6	Microscope	No	No
Hartline et al. [39]	Determine how type of washing-machine effects emissions of fibres from new and mechanically aged jackets and sweaters	Front and top	1	No	30–40	N/A (top) /1200 (front)	30 (top) /24 (front)	Five different types of jacket (2 fleeces)	Polyester, polyamide	333 & 20	No	A balance directly measured each membrane after filtration and the approximate initial mass of all membranes was inferred using an average reference ratio.	Image J
Napper & Thompson [41]	Test effect of polymer, temperature and detergent on the release of fibres from polyester, polyester-cotton blend and polyacrylonitrile fabrics.	Front	1	Yes/No	30& 40	1400	75	Patches of sweaters	Polyacrylonitrile, polyamide and polyester	25	Microscope and equation: indirect estimates	Microscope and equation: indirect estimates	Microscope (approximate estimates)
Pirc et al. [38]	Examine emissions of fibres from domestic washing of polyester fleece	Front	1	Yes/No	30	600	15	Blankets	Polyester	200 (& 2–3 in two samples)	No	Balance directly measured each membrane before and after filtration.	ImageJ
Hernandez et al. [53]	(1) Determine how variations in the construction (fibre and knit) and washing of textiles affects emissions of fibres, (2) develop standardized protocols to quantify release of fibres in terms of length and total mass, and (3) test how textile-construction and washing- conditions alter emissions of fibres from domestic washing conditions.	Centrifuge	1 but has multiple vessels in a block	Liquid and powder	25, 38–42, 60, 80	38–42	45, 60, 120, 240, 480	Patches	Polyester	0.45	Microscope	Microscope and equation: indirect estimates	Microscope
Carney Almroth et al. [42]	Quantify fibres shedding from fabrics that vary by polymer, knitting techniques, state (new vs damaged items) and washing procedures (use of detergent and number of washings)	Centrifuge	1 but has multiple vessels in a block	Yes/no	60	N/A	30	Fabric swatches with different gauges and knit	Polyacrylonitrile, polyamide, polyester	1.2	Microscope	No	No
De Falco et al. [43]	Compared emissions of fibres from different plastic fabrics washed in domestic (light, heavy, oxy, bleach, softener) and industrial (alkaline detergent, sapoigeienbacto, oxitex) detergents and water (hard, distilled) and balls (10, 20)	Linitest (URAI S.p.A., Asago, Italy)	1 machine but has multiple vessels but a blocked design seems to have been used	Yes/no	40,75	N/A	45, 60	Patches	Woven and knitted polyester and woven polypropylen	5	Microscope	Microscope and equation indirect estimates	ImageJ
McIIwraith et al. [44]	Test that there would be less microfibres in effluent from washes using Cora balls or external Lint-luver filters.	Top	1	No	16	120–660	30	Blanket	Polyester	10	Microscope	Balance directly measured each membrane before and after filtration.	ImageJ

There are currently no standardized methods or metrics for assessing the effectiveness of washing-machine filters or the emissions of fibres from clothes to sewage; however, a number of studies have shed light on these important topics (Table 1). Several studies have included garments that vary by type (i.e. blanket, fleece, shirt, jumper, sweater, jacket), polymer (i.e. polyester, polyacrylonitrile, polyamide, polypropylene, cotton), construction (i.e. knit, gauges) and/or their entirety, from whole garments [4, 38–40] to swatches (i.e. patches) of cloth or fabric [41–43]. These garments were washed using domestic washing-machines [4, 39] or industrial centrifuges [42–43] using different temperatures (30, 40, 60 °C), speeds (600, 1200, 14000 rpm) and durations (15, 30, 48, 45, 48, 75, 90 min). Experiments that mimic real-world conditions, for example with whole garments [38, 39, 44] and washed in multiple domestic appliances according to their care-labels [4] arguably present more translatable results than experiments with swatches washed in a single washing-machine [41], or a centrifuge designed to test the ability of fabrics to retain their colour [42, 43]. In addition, more accurate and larger quantities of micron-sized fibres have been collected by using techniques that filter the effluent using membranes with small pores (0.45–1.6 μm) [4, 42], in contrast, less accurate and/or smaller quantities of micron-sized fibres have been collected by filtering the effluent using membranes with larger pores (10–333 μm) [38, 39, 41, 44] and/or using a microscope to estimate the abundance and mass of fibres [38, 44]. Furthermore, statistical tests vary from powerful parametric [41] to less powerful non-parametric tests [43] that are less able to cope with heterogenous variances [45]. Previous work has investigated the performance of filters on a washing-machine [44] in relation to the masses, numbers and lengths of large polyester fibres in effluent emitted from washing blankets with and without a single external filter (Lint Luv-r) and replicate internal filters (Cora Ball). Using a single top-loader appliance, this study found that replicate internal filters reduced the mass of fibres in the effluent by 5% and a single external filter (which was repeatedly used) reduced the mass of fibres in the effluent by 80%. While these experiments help build our understanding of the efficiency of filters, further work is needed to increase the precision, generality and realism of the experiments by using replicate appliances, external filters, and garments made from different polymers and washed according to their care-instructions. This is important because the physical and functional properties of these products will vary due to processes occurring during and after manufacture. Therefore experiments with greater levels of replication (as is well known for all surveys and experiments [45–49]) are needed to determine whether effects of the filters actually arise over and above natural variations in fibre-emissions that are excluded by experiments lacking replicate external filters, appliances and garments made from different polymers. To improve our understanding, further work is needed to gather data about the ability of filters to retain and emit fibres so that the proportion of the total fibres retained by the filter can be determined for each wash. As such, the field of research would benefit from additional experiments in which replicate garments made from natural and synthetic fibres are independently washed in replicate appliances with and without replicate filters (according to clothing care-instructions) and fibres trapped and not trapped by the filter are quantified and analysed statistically.

Here we developed and tested a novel protocol to assess the capacity of two commercially available washing-machine filters with either mili- or micro-meter-sized pores to trap and reduce emissions of fibres by mass (here after referred to as debris) from clothing (not blankets or smaller patches of fabric) to sewage. In order to provide translatable and rigorous testing of the washing-machine filters our experiments (i) washed whole garments in domestic appliances; (ii) used common garments made from natural and synthetic fibres washed according to their care instructions (e.g. cycle, temperature, speed, duration); (iii) used replicate and independent garments and filters to improve the independence of data; (iv) used multiple domestic washing-machines across brands so that results can be more easily generalised; (v) removed fibres from effluent using membranes with smaller pores that catch all of the micron-sized fibres of interest; (vi) quantified fibres trapped and not trapped by the filter in terms of their actual and relative mass; (vii) used parametric statistical analyses to improve certainty about how washing-machine filters with different sized pores affect emissions of fibres.

## Materials and methods

### Experiments with washing-machines

**Fig 1:**
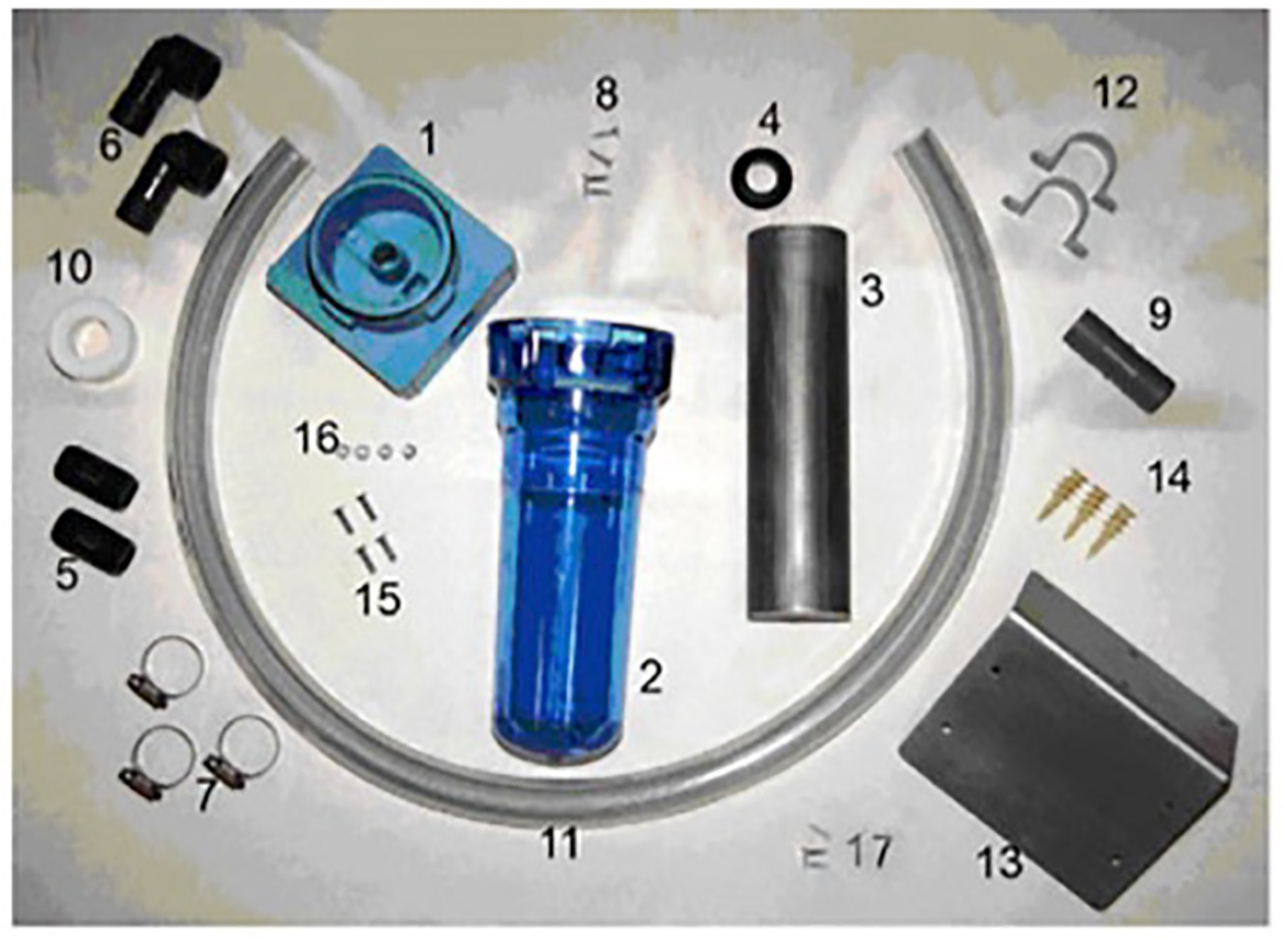
Composition of the washing-machine filter. This includes a filter-head (1), filter case (2), stainless steel filter of either 2 mm or 150 μm pores (3), rubber grommet (4), 19 x 51 mm pipe nipples (5), 19 mm female x 25 mm barbed hose with a 90° elbows (6), stainless steel clamps (7), wood-screws (8), 25 mm barbed tube (9), polytetrafluoroethylene tape (10), 25 x 1000 mm hose (11), 25 mm conduit clamps (12), metallic angled mounting bracket (13), drywall anchors (14), 19 mm machined screws (15), machined screw nut (16), 19 mm wood screws 19 mm. This picture was provided with permission from B. Jollimore at www.environmentalenhancements.com.

**Fig 2:**
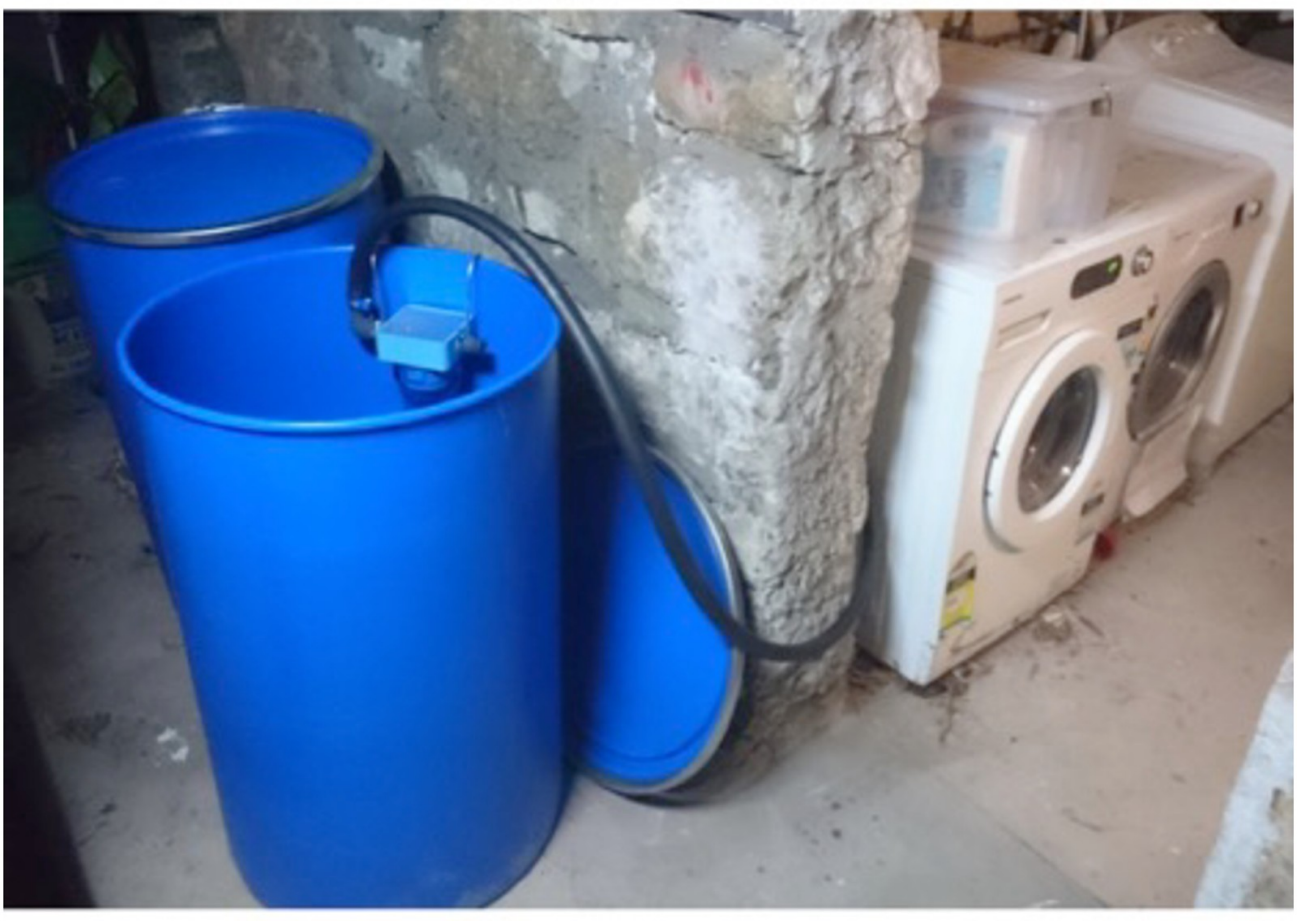
Filters connected to domestic appliance and 200 L polyethylene drum.

**Fig 3:**
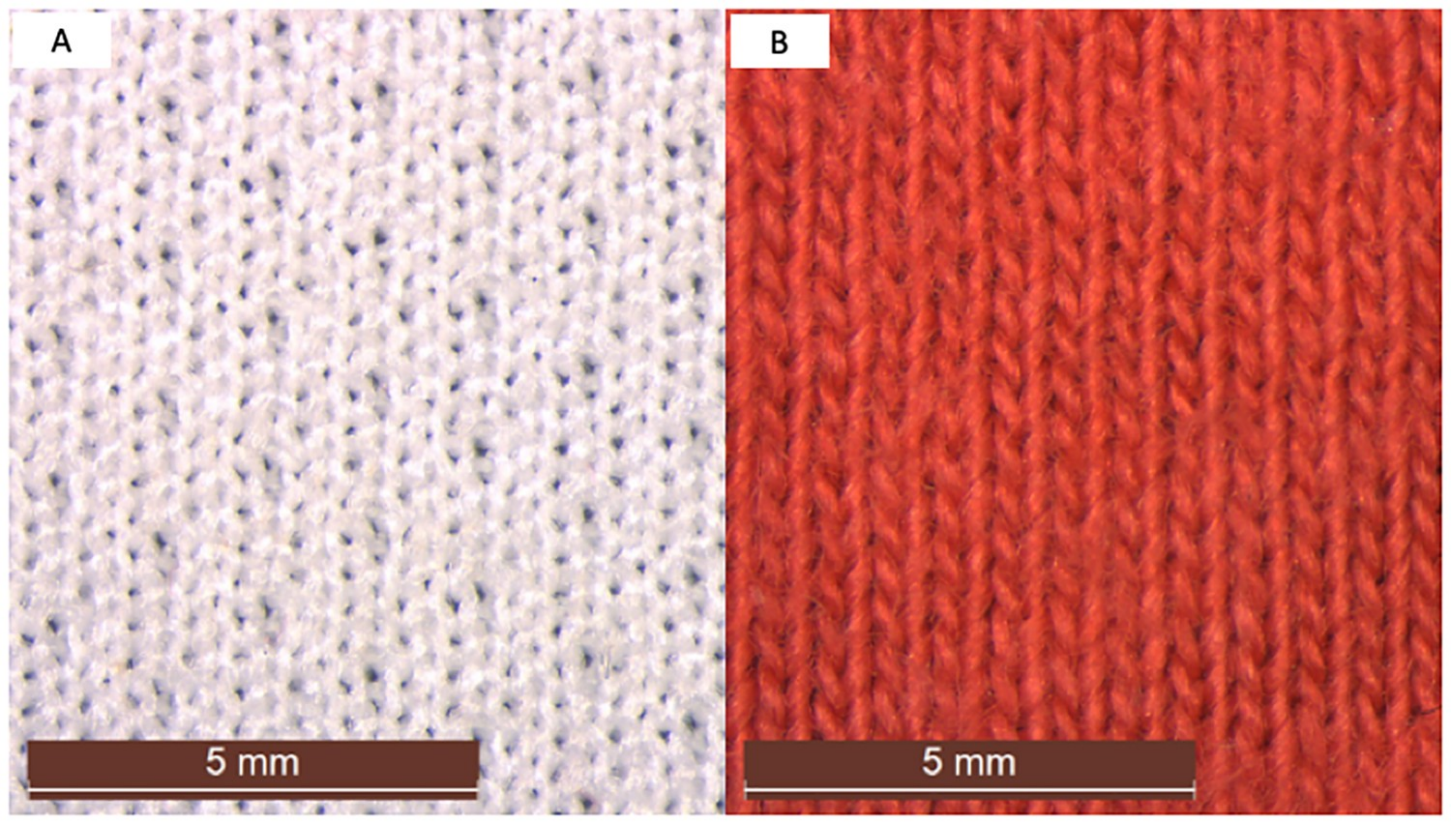
Physical structure of garments used in experiments. Polyester t-shirts were composed of a polyester staple fibre with a hexagonal cross-section arranged at a density of 18 rows and 25 columns per cm fabric with a mock eyelet double jersey knit. Cotton t-shirts were composed of a staple fibre with a “bean” shaped cross-section arranged at a density of 15 rows and 20 columns per cm fabric with a single jersey knit.

**Fig 4:**
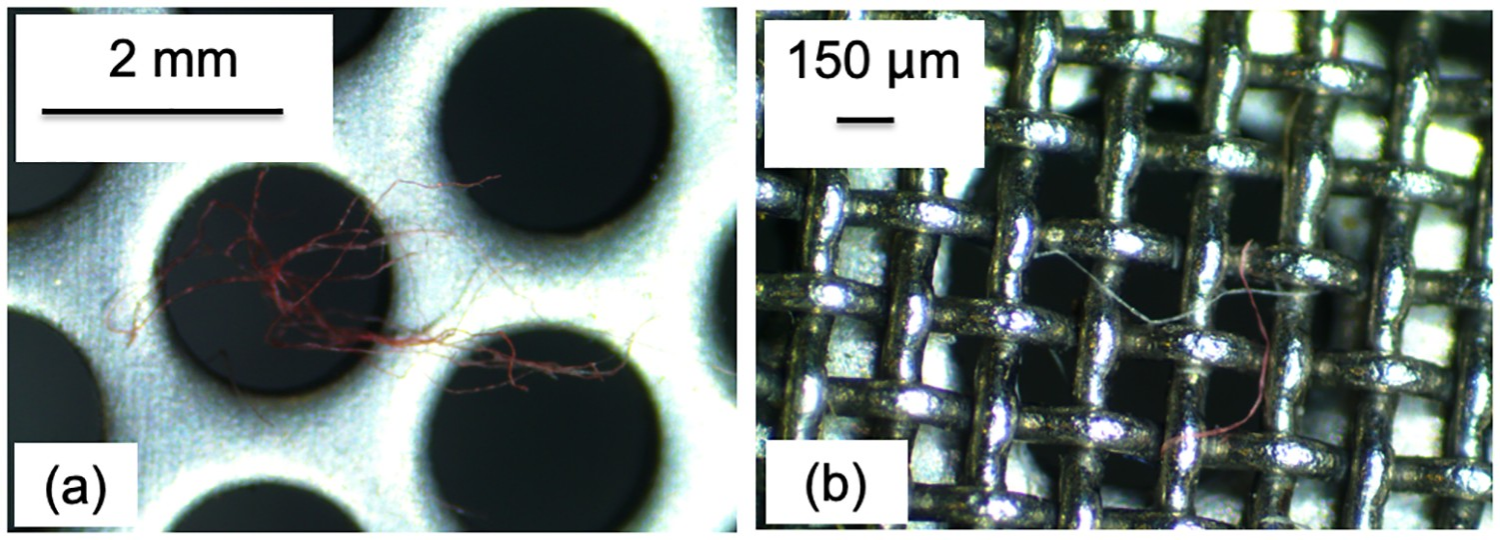
The metallic cores of the filter with 2 mm (a) and 150 μm sized pores (b).

To test the capacity of a commercially available washing-machine filter (Lint-luver [26]) to trap and reduce emissions of clothing debris to sewage we completed two experiments with polyester or cotton t-shirts washed in front-loader washing-machines. Here our primary aim was to test the efficiency of filters (within budgetary constraints) using the garments, appliances, types of wash and filters that the public uses. Polyester and cotton were chosen because they are the most common polymers used to make garments with over 46 and 24 million tons of each polymer respectively used each year [50]. T-shirts were chosen because along with singlets they represent 8% (US$68 billion) of apparel sold in North America, Europe and Australia [51]. Our experiments used Gildan ‘paprika’ cotton t-shirts (Gildan Activewear, Canada) and Sportz white polyester t-shirts (Sportz, Australia) (Fig 1) and these are purchased by the public and washed by the public according to their care-labels. As such we chose established settings for polyester (30°C, 600 rpm) and cotton (40 °C, 1000 rpm) garments from the instructions of the multiple front-loaded washing-machines (Samsung Bubble Wash, Samsung WF7708N6W1, Omega OWD 6000 WA, Bosch Classixx, Miele WT2670; Figs 2 and 3) at 5 separate domestic locations across New South Wales, Australia. In each location, there were 3 treatments, done at random, consisting of washes with no filter, washes with a 2 mm pore-sized filter and washes with a 150 μm pore-sized filter and there were separate filters for each household (Fig 4). Whilst we acknowledge that the results may vary if different machines or textiles were chosen, our protocol is in keeping with protocols for experimental design and statistical analyses [45]. These require an appropriate experimental setting (e.g. domestic) and units (machines, filters, garments) that represent as much as possible the random subset of those used by the public and required by the statistical test. All filters were provided for free by Environmental Enhancements and the broader 2 mm pore-sized filter was originally sold by this manufacturer while the finer 150 μm pore-size was a modification they made after discussions we had with them. In each wash, 3 new and unwashed t-shirts were washed using the programmes for cotton or synthetic clothes. To help improve the independence of the data, 3 empty washes were used to remove debris between each test wash. In keeping with previous studies [3, 34], detergent was not used because it would confound measurements of mass. The absence of detergent represents an ongoing problem for the ‘realism’ of all studies assessing emissions of fibres from clothes by mass and cannot be remedied until a method is developed to remove or quantify the mass of the detergent from the mass of the fibres/yarns.

### Quantification of debris from clothing

To quantify fibres discharged from each wash the effluent was collected in a 200 L polyethylene drum (www.peopleinplastic.com.au) and the filter-case. The volumes of effluent in the filter-case and drum were then determined using electronic balances (Mettler Toledo MS). The total volume of water in the filter-case was collected in metallic flasks and then effluent in the drum was stirred for 30 seconds (to help ensure the fibres were evenly distributed) and then triplicate samples of water (each 100 mL for cotton or 200 mL for polyester) were collected in more metal flasks. Drum and filter-case samples were then vacuum filtered onto 47 mm glass fibre filters (Pall Corp. Type A/E 1 μm) and placed in petri dishes to determine the total mass of debris. The metallic core of the filter was then removed from the filter-unit, wrapped in aluminium foil, dried at (50°C, 48 h) in an oven (Labec) and then the mass of debris on the metallic core quantified using an electronic balance (Mettler Toledo MS). This temperature is cooler than those used to make garments (M. Todesco, Textile Engineer at University of New South Wales, pers. com.) and even if the temperatures of the washes did evaporate compounds from the fibres and yarns, it would occur for all treatments and experimental units so is less likely to affect the pattern of results. To reduce cross-contamination by fibres we used cleaned and new containers, petri dishes, laboratory coats and we filtered the effluent in a glass cabinet inside a large closed polyethylene tent. For each wash, data about the volumes of effluent and the mass of debris in the drum and filter-cases were combined to determine the total mass of debris in the effluent of the drum and filter-case. Then the debris trapped on metallic core of each filter was also determined. This provided replicate data about the debris for each wash with cotton (n = 3) or polyester (n = 5) clothes in terms the quantities of debris entering sewage, the quantities of debris prevented from entering sewage by the filter, and the net-reductions (%) in debris entering sewage. Each metric for each polymeric material (i.e. cotton or polyester) was analysed using a balanced one-factor analysis of variance (Fixed: 3 levels of filtration) with 3 replicates for cotton and 5 replicates for polyester followed by post-hoc SNK tests (GMAV, EICC, University of Sydney).

### Use of water

The total volume of water used in each wash was analysed using two-factor analysis of variance with 3 replicate values from each wash and for polyester 3 were randomly chosen from the 5 available. Here the with the first factor ‘Polymer’ (Fixed with 2 levels: cotton and polyester) and the second factor ‘Filtration’ (Random with 3 levels of filtration) followed by post-hoc pooling and SNK tests [45].

## Results

### Mass of debris in effluent: Effect of filters

**Fig 5:**
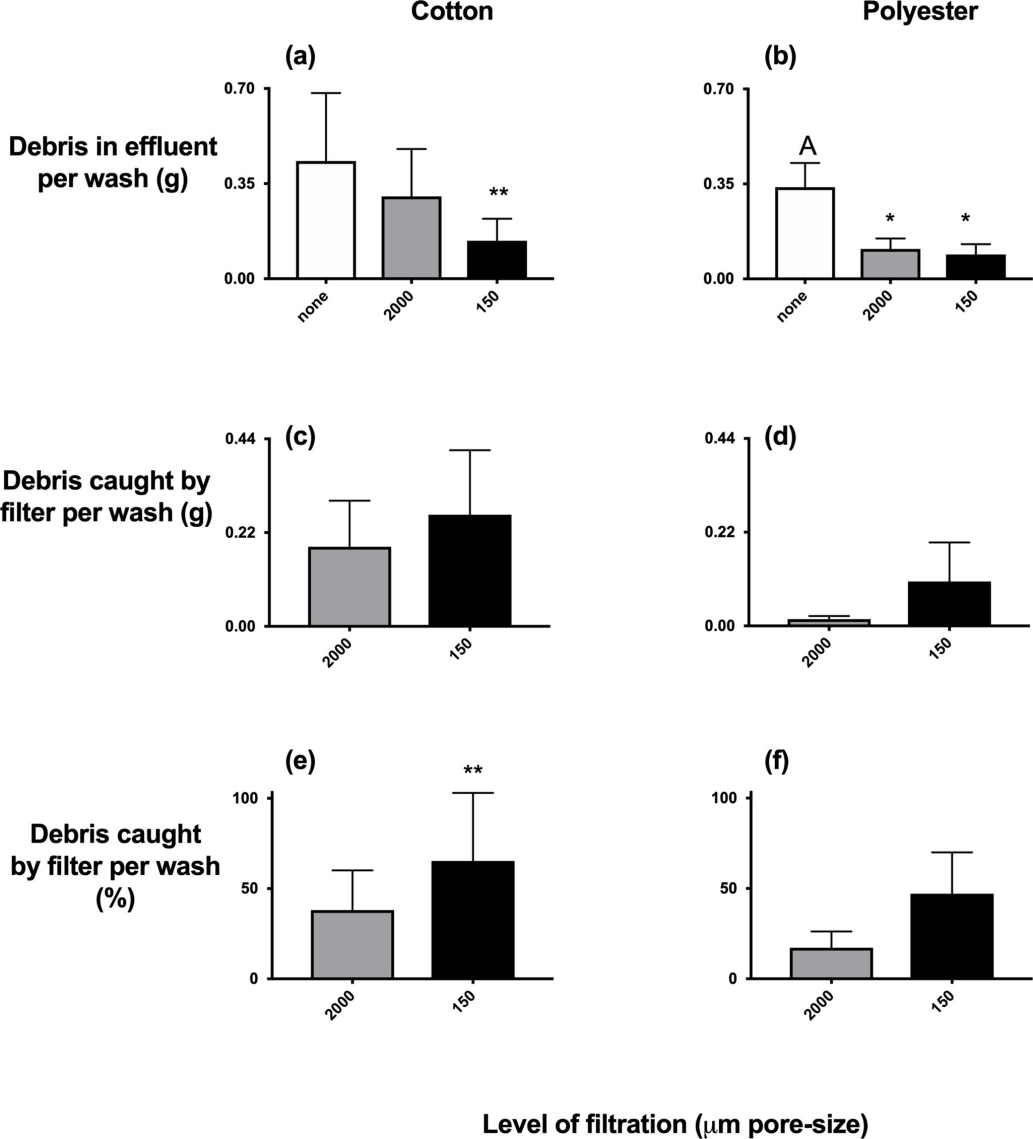
Emissions of debris from washing cotton and polyester t-shirts in appliances with and without filters. Significantly different groups from ANOVA and SNK tests are indicated at P<0.05* and P<0.01**.

Adding micron-sized filters to washing-machines reduced the mass of cotton in their effluent by 67% compared to appliances with no filters (F_2,6_ = 11.69, P<0.01), while millimeter-sized filters had no significant effect (Fig 5a). A different pattern was observed for experiments with polyester t-shirts (Fig 5b). Here, appliances fitted with either micron or millimeter-sized filters emitted between 65% (millimeter-sized filter) and 74% (micrometer-sized filter) smaller masses of polyester compared to appliances with no filters (F_2,12_ = 5.20, P<0.05).

### Mass and proportions of debris trapped by filters

Both types of filters caught similar masses of cotton (t = 1.61 df = 4, ns) and polyester (t = 1.24 df = 8, ns) with no difference between the two types of filter (Fig 5c and 5d). When the masses retained on the filter were standardized to the total masses of debris emitted from the t-shirts (Fig 5e and 5f), micrometer-sized filters caught significantly larger proportions of cotton (t = 4.80, df = 4, P<0.01) but not polyester (t = 1.58 df = 8, ns).

### Use of water

**Fig 6:**
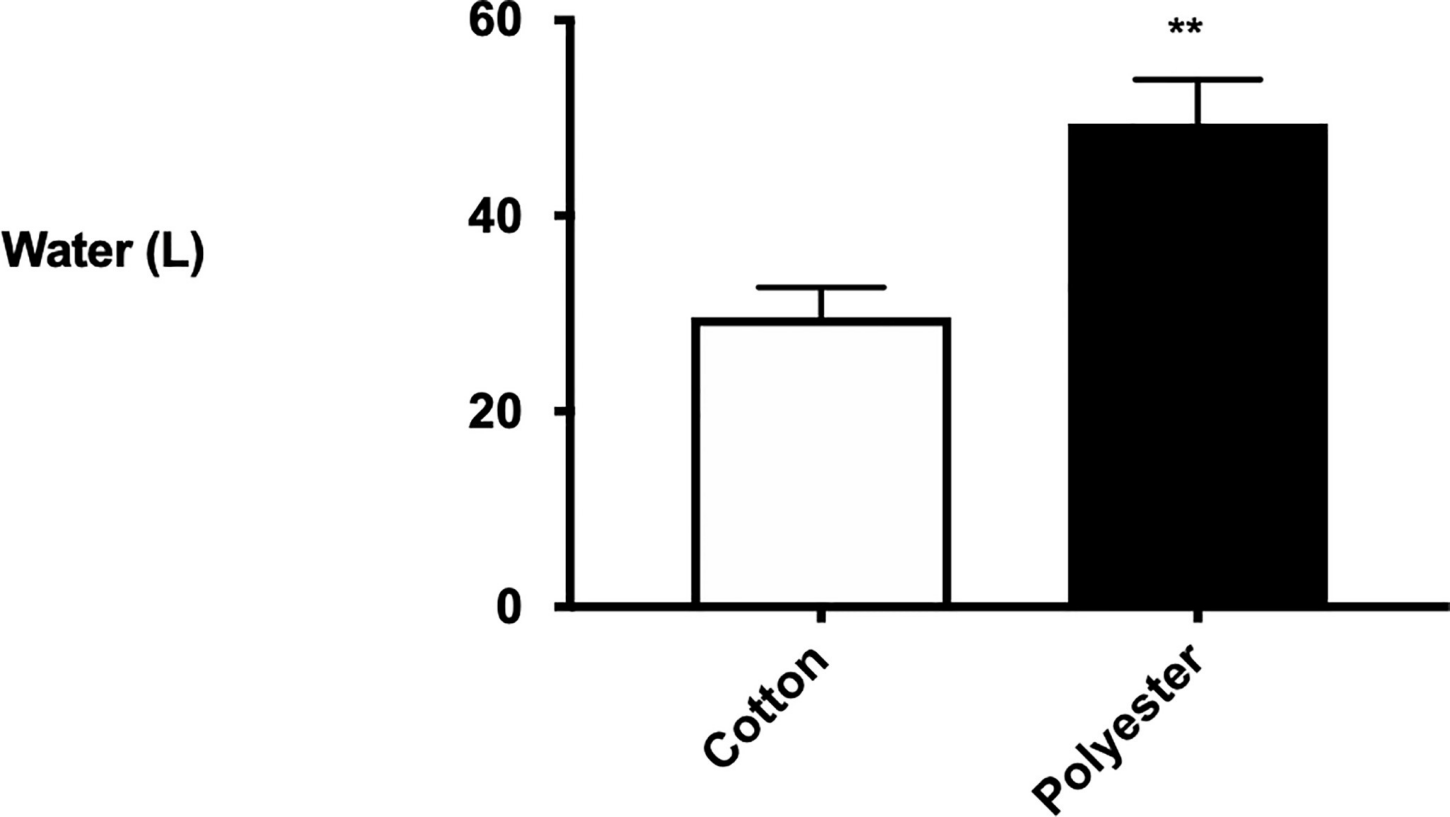
Emissions of water from washing cotton and polyester t-shirts. Significantly different groups from ANOVA and SNK tests are indicated at P<0.01**.

Washing synthetic garments (49.4±4.52 L per wash) used 167% more water than washing cotton garments (29.64±4.52 L per wash) (F_2,12_ = 5.20, P<0.01; Fig 6).

## Discussion

Our study is the first domestic experiment to compare the capacity of replicate washing-machines and filters to reduce the emissions of natural and synthetic clothing fibres to sewage. Here we show for the first time that adding filters to a range of domestic washing-machines significantly reduces the mass of cotton and polyester in effluent by more than 65% and that the performance of filters varies depending upon the pore-size of the filter and the type of wash/polymer. Our use of replicate garments (made from natural and synthetic fibres) domestic appliances, filters and the quantification of the masses and proportions of micron-sized fibres trapped both in the filters and in the effluent builds on previous laboratory work [44] to provide more translatable and rigorous testing of the washing-machine filters. Evidence from these studies builds a picture about the efficiency of external filters that if systematically and critically interpreted can ensure consumers and companies can make useful and certain actions to reduce the quantity of clothing fibres washed into the ecosystem.

### Identifying features of clothes and filter that reduce emissions of fibres to sewage

Our study showed filters with smaller pores trapped larger masses and proportions of fibres, however, the significance of patterns varied in relation to the type wash/garment. Therefore further work is needed to distinguish the features of clothing and/or the wash that cause these differences in the performance of the filter. Because synthetic and natural fibre clothing vary by polymer and in their construction (e.g. length and width of fibres/filaments, the number and type of weave, knit, stiches, hems) more complicated multifactorial experiments [45] than have previously not be done [44, 52] are required to determine with certainty how these single and combined features affect emissions of fibres. Microscopic observations of the garments using a dissecting light microscopy revealed that the polyester t-shirts were composed of filament fibres with a hexagonal cross-section that were stitched together at a density of 18 rows and 25 columns per cm with a mock eyelet double jersey knit. In contrast the cotton t-shirts were composed of staple fibres with a bean shaped cross-section that were stitched together at a density of 15 rows and 20 columns per cm in a single jersey knit (Fig 1). Because fabrics made from cotton can be stronger and more durable than fabrics made from polyester [53], cotton clothing is washed using smaller volumes of warmer water with more agitation, while polyester clothing is washed using larger volumes of cooler water with a slower spin to avoid stretching the synthetic fabric [54–55]. This was supported by our experiments which showed larger volumes of effluent discharged through filters from washes with polyester t-shirts than those with cotton t-shirts. To unravel what features of clothing and the wash alter the performance of the filter will require more complicated multifactorial experiments in which the physical and chemical properties of fabric and the wash are manipulated.

In our study limited resources meant we could only estimate the mass of fibres. We considered mass as the most relevant metric for assessing the efficiency of filters because it can be related to the total mass of garments and the polymers used in manufacture. Even so further work would also benefit from characterising emissions of fibres by size, shape and number, as it would enable tests to determine if variations in the significance of the patterns observed by the mass of fibres in the effluent and trapped on filters (total and proportions) could be generalised to numbers and particular sizes and shapes of fibres in the effluent and trapped on filters. This would yield useful information about the types of fibres trapped by filters which would enable engineers to reengineer features of the filter so scientists can test whether they can trap a broader range of shapes and sizes of fibres. Whilst it is tempting to use filters with even smaller pore-sizes, these could alter the efficiency of appliances and/or damage the motor, pump, valves, drum, agitator, paddles, electrical circuits and the heating elements of appliances. So any changes to the design of the filters needs to be based on experiments that provide robust information about how features of the filter interact with the type of appliance, wash and garment to affect emissions of fibres and the performance, durability and safety of appliances.

The limited budget and number of independent filters also meant it was not possible to test potential sources of variation attributable to the machines and further studies should assess this by having replicate washes of each treatment done using each machine. The importance of replication and statistical tests are well established [45–49] and experiments (domestic and laboratory) would benefit from comparisons amongst factors of interest involving more replication and parametric statistical tests. Replication can, however, be expensive and our budget limited our work to 3 garments per replicate load. Whilst this is more representative of a typical wash than swatches of fabric or single garments, experiments could be more representative if the loads of washing used were informed by domestic surveys of washes and there is sufficient resources to provide independent loads of garments to be experimentally washed. In keeping with the stewardship of other products [56] robust developments often only occur when there are specific changes in efficiency with which companies, scientists, engineers and policymakers think and interact. Where there has been sufficient resources, academia, industry and government have developed and ran standardized tests (using standardized types and quantities of fabric, detergents and organic matter) to assess the durability [57] of washing-machines and how much energy and water they use [58]. Currently one appliance is independently tested and if the results are not equivalent to the values declared by industry (within a certain tolerance range) than another three appliances of the same model are taken from the market and tested [59]. Interdisciplinary systematic and critical reviews of existing studies on washing-machines and the use of filters would help determine if these tests could be improved to make general and certain conclusions by including more representative wash-loads, levels of replication and statistical tests.

### Testing the capacity of filters to mitigate environmental contamination

While it is tempting to combine data from small-scale experiments and surveys to estimate the potential environmental impact of a whole city using washing-machine filters [44], such estimates must be done with caution. Sensible extrapolations will require structured surveys and experiments [45, 48] in the same city to collect robust data, throughout the year, about the (i) composition of clothes washed per resident; (ii) capacity of washing filters attached to these appliances to reduce the stocks of fibres to and from facilities treating sewage; (iii) the residence-time of fibres in different parts of facilities treating sewage. Given that the type of season, and weather will affect what clothes are washed and how long their fibres are retained in sewage treatment plants, we urgently require better surveys, experiments and models to determine with certainty the capacity of filters to alter environmental emissions of fibres. Furthermore, experiments are also needed to test the efficiency of different methods and frequencies of cleaning, including how they affect the types and quantities of fibres that humans, wildlife and habitats are exposed to and the impacts they may cause.

## Conclusions

Our study provides insights from replicated and more realistic testing of two washing-machine filters used on whole garments of clothing. This information contributes to our growing understanding of the critical need for, and effectiveness of, technologies that can reduce contamination and pollution by fibres and yarns. While products to mitigate pollution are already available for purchase in some countries, insufficient peer-reviewed information is available to understand the effectiveness of these products. We provide evidence that one filter-design may more than halve emissions of fibres to effluent but the metric, pore-size and polymer chosen can affect the ability of filters to reduce domestic emissions of fibres to sewage. Given that three different measures of the performance of a filter resulted in different patterns of results for each type of garment has important implications for this field of research and innovation. Previous studies used one top-loader machine to wash replicate garments with and without one of the same models of external micron-sized filter [44]. Through this the study showed that the filter reduced the mass of polyester fibres in the effluent by 80% which is larger but similar to our value of 74%. Our study, however, suggests that the efficiency of filters should be tested using different types of garments with detailed measurements of the quantities of fibres trapped on filters and in the effluent. This would allow the efficiency of the filter to be known in terms of the proportion of fibres trapped by the filters. In our experiment neither cotton or polyester fibres accumulated in significantly greater masses on either sized filter. Both sized filters significantly reduced the mass of polyester in effluent, whilst for cotton only the addition of a micron-sized filter to washes significantly reduced the total mass of fibres in effluent but not when the proportion of fibres on filters relative to the effluent was tested. Taken together these results suggest that domestic experiments may yield larger variations than more standardised laboratory experiments. Whilst that might discourage some from using domestic experiments, the variation represents our uncertainty about the efficiency of the filter in the real world and this can be dealt with if the researchers have the necessary budget and expertise in designing and analysing multifactorial experiments [45, 48]. Given the increasing numbers and types of clothes, polymers, appliances and filters used by the public, our work shows more expensive and complex (e.g. more treatments) and powerful (e.g. greater levels of replication including on the same appliance) surveys and field experiments are needed to determine with certainty the features of clothes, washes, and/or filters that reduce emissions of debris from clothes to sewage.

## Supporting information

S1 Data Raw data for fibres emitted and retained by filters. (XLSX)
